# The MAS4AI framework for human-centered agile and smart manufacturing

**DOI:** 10.3389/frai.2023.1241522

**Published:** 2023-09-28

**Authors:** Aleksandr Sidorenko, William Motsch, Michael van Bekkum, Nikolaos Nikolakis, Kosmas Alexopoulos, Achim Wagner

**Affiliations:** ^1^Deutsches Forschungszentrum für Künstliche Intelligenz GmbH, Kaiserslautern, Germany; ^2^Nederlandse Organisatie voor Toegepast Natuurwetenschappelijk Onderzoek, The Hague, Netherlands; ^3^Laboratory for Manufacturing Systems and Automation, Department of Mechanical Engineering and Aeronautics, University of Patras, Patras, Greece

**Keywords:** Artificial Intelligence, Asset Administration Shell, human-centered AI, multi-agent system, Human Digital Holon, self-configuration, shared assembly

## Abstract

Volatility and uncertainty of today's value chains along with the market's demands for low-batch customized products mandate production systems to become smarter and more resilient, dynamically and even autonomously adapting to both external and internal disturbances. Such resilient behavior can be partially enabled by highly interconnected Cyber-Physical Production Systems (CPPS) incorporating advanced Artificial Intelligence (AI) technologies. Multi-agent solutions can provide better planning and control, improving flexibility and responsiveness in production systems. Small modular parts can autonomously take intelligent decisions and react to local events. The main goal of decentralization and interconnectivity is to enable autonomous and cooperative decision-making. Nevertheless, a more efficient orchestration of various AI components and deeper human integration are required. In addition, global behaviors of coalitions of autonomous agents are not easily comprehensible by workers. Furthermore, it is challenging to implement an Industry 4.0 paradigm where a human should be in charge of decision-making and execution. This paper discusses a Multi-Agent System (MAS) where several software agents cooperate with smart workers to enable a dynamic and reconfigurable production paradigm. Asset Administration Shell (AAS) submodels hold smart workers' descriptions in machine-readable format, serving as an integration layer between various system's components. The self-description capability of the AAS supports the system's adaptability and self-configuration. The proposed concept supports the plug-and-produce functionality of the production modules and improves human-machine integration in the shared assembly tasks.

## 1. Introduction

The increasing advancements in automation and Artificial Intelligence (AI) improve efficiency, accuracy, and consistency in modern production systems (Felsberger et al., 2022). Automation of routine tasks allows employees to tackle more creative activities, increasing their work satisfaction (Kolade and Owoseni, 2022). Robots are already performing repetitive and hazardous tasks, while the latest AI solutions extend this transformation to the white-collar service sector. Automation and AI allow machines to accurately perform tasks previously done only by humans (Spring et al., 2022). This increases product quality by eliminating the risk of human error. However, humans remain the organization's most flexible and adaptable elements. Human knowledge, experience, innovative thinking, and creativity are the main factors of success for an organization (Azeem et al., 2021). Hence, it becomes critical for the factories to manage their digital transition in a balanced and inclusive way toward their workers.

Effective and autonomous control in smart manufacturing includes the successful integration of advanced AI technologies in the existing production processes and equipment (Arinez et al., 2020; Jan et al., 2022). Considering active control as a key requirement in this context, data processing at the edge along with the offline decision-making capabilities are necessary, as the connection to the cloud may be intercepted or lost. Such complex production systems, distributed and concurrent in nature, are expected to be flexible and adaptable. They will operate at increased speeds, and exhibit better robustness, scalability, and reusability. For the aforementioned reasons, cloud-based or hybrid platform solutions do not seem suitable. In contrast, the software agent technology is one approach for the development of interoperable software applications in distributed, heterogeneous and even unreliable environments (Tello-Leal et al., 2014), such as modern production systems. Multi-Agent Systems (MASs) are suggested as suitable for implementing Industry4.0 components (Sakurada and Leitão, 2020).

Though MASs are very common in the field of human-assistance control (Marks et al., 2018), there are some limitations in recent approaches to applying MASs in manufacturing systems. For example, the user interfaces are mostly allocated to the system level and not to the specific agents, which could provide their own interfaces if necessary. Moreover, the coordinating agents are mostly centralized in their architectures, which can hinder modularity and flexibility. Furthermore, agent-based assistance systems often provide only a low level of automation, i.e., the computer offers a complete set of decision/action alternatives without narrowing down the selection or supporting the action. Additionally, the number of supported levels in the automation pyramid is very low and the interoperability with existing industrial software frameworks is very limited due to missing or partially implemented industrial standards (Lu et al., 2020). Summing up, the seamless integration of human factors in MAS architectures and the implementation of such systems are still very challenging.

In this work, we focus on the following questions. How can the existing MAS-based approaches for reconfigurable manufacturing systems be improved under consideration of Industry 4.0 concepts to ensure collaboration among heterogeneous production assets, including humans and AI components? How to make human characteristics available to the production system in order to improve its interaction with the workers? How to realize functional decomposition in distributed control architectures for effective sharing of tasks between humans and machines? By addressing these questions, we contribute to the transition to a human-centric and resilient manufacturing paradigm.

The paper presents a multi-agent framework that extends the existing approaches for designing reconfigurable modular production systems by proposing a concept of Human Digital Holon for improving human-system integration. The proposed framework is based on the RAMI4.0 reference architectural model for the effective utilization of the Industry4.0 enabling technologies (Frysak et al., 2018). The Asset Administration Shell (AAS) is used for the digital representation of all the assets, as well as the MAS elements. This increases interoperability between the system's components and potentially lowers the integration efforts. The human-system integration is achieved by modeling various human aspects as AAS submodels and by augmenting human behavior with a digital holon. The framework is conceptualized in a prototype and is currently being tested in several industrial use-cases, though without providing a final evaluation yet. Two scenarios described in the paper support the main findings and show how humans can be integrated into the shared human-machine tasks.

This paper is structured as follows. A literature review is presented in Section 2. It gives some theoretical foundations of the proposed framework and analyzes the current approaches for enhancing systems reconfigurability and integration of humans into production. The topics of multi-agent and holonic manufacturing systems are discussed in Section 2.1, Digital Twins, Cyber-Physical Systems and Holons—in Section 2.2, service-oriented applications in the context of industrial automation domain—in Section 2.3. Finally, Section 2.4, outlines recommendations and challenges for the design of human-centered Cyber-Physical Systems, as well as discusses the current research gaps and the main focus of this study. The key concepts for human-centered and smart manufacturing, which are central to our framework, are introduced in Section 3. Semantic modeling and the asset administration shell for a smart worker are explored in Sections 3.1 and 3.2, respectively. The concept of a Human Digital Holon is presented in Section 3.3. Section 4 describes the proposed framework, which is outlined in Section 4.1. Section 4.2 elaborates on how to build a holon in the context of the framework. In Section 4.3, we discuss the testing aspects of the system's prototypes. Section 5 describes two case studies of integrating a smart worker into modular reconfigurable production lines using our approach. Section 5.1 gives the testbed description. The first scenario in Section 5.2 shows how the self-description capability of the AAS can support plug-and-produce and self-configuration functionalities of the system. The second scenario in Section 5.3 explores the use of holons and Behavior Trees for human-robot coordination in the shared assembly tasks. In Section 6, we discuss the proposed framework in the context of reconfigurable production and human-system symbiosis, including some challenges and open questions. Section 7 concludes the work and outlines the next steps of the current study.

## 2. Literature review

The proposed framework builds upon the RAMI4.0 reference architecture (DIN SPEC 91345, 2016) and the core enablers of the Industry 4.0, such as Cyber-Physical Systems, multi-agent and service-oriented architectures and semantic interoperability. In this section, we give some theoretical foundations, as well as analyze current approaches for enhancing manufacturing systems reconfigurability and human system integration. In Section 2.1, we review the agent- and holon- based approaches in manufacturing, and how these concepts influence our architecture. In Section 2.2, we define Cyber-Physical Systems and Digital Twins, and their role in the modern Cyber-Physical Production Systems. Section 2.3 reviews the Service-Oriented Architectures and how the skill-based approach can be used to encapsulate complexity and separate implementation from the business logic. Lastly, in Section 2.4, we examine the current approaches for incorporating humans into production systems.

### 2.1. Multi-agent and Holonic manufacturing systems

Over the years, many definitions of agents have been proposed, focusing on different aspects of the concept. Russell and Norvig (2020) give a more CPS-oriented definition of an agent as “anything that can be viewed as perceiving its environment through sensors and acting upon that environment through actuators”. Leitão (2009) focuses on autonomy and interacting capabilities of agents: “An autonomous component that represents physical or logical objects in the system, capable to act in order to achieve its goals, and being able to interact with other agents, when it does not possess knowledge and skills to reach alone its objectives”. A Multi-Agent System (MAS) is a federation of (semi-)autonomous problem solvers that cooperate to achieve their goals and also the global system's goal. To succeed, they rely on communication, collaboration, negotiation, and responsibility delegation (Leitao and Karnouskos, 2015). As a general technology focused on intelligence, autonomy, and cooperation, MAS is widely applicable in the manufacturing domain. Over several decades of applying multi-agent approaches in industrial applications, many MAS architectures have been proposed. Leitao and Karnouskos (2015), Leitão et al. (2016), and Cruz Salazar et al. (2019) give good overviews of some of them.

Holonic manufacturing systems (HMS) is a manufacturing paradigm proposed at the beginning of the 1990s as an attempt to improve the ability of manufacturing systems to deal with the evolution of products and make them more adaptable to abnormal operating conditions (Giret and Botti, 2004). This paradigm suggests that besides autonomy and interaction, which the classical MASs focus on, manufacturing systems will continue to need hierarchical structures to lower complexity and resolve conflicts between different agents (Leitão, 2009). A holon is an autonomous, intelligent, and cooperative building block of a manufacturing system that serves transformation, transportation, or other industrial tasks (Van Leeuwen and Norrie, 1997). A manufacturing holon consists of an information processing part and can also have a physical processing part. A holarchy, being a system of holons, defines the rules for interaction between holons and thus limits their autonomy. Each holon can be simultaneously a part of several holarchies and be a holarchy itself (Christensen, 1994). This enables very complex and flexible control structures, also called flexible hierarchies. Christensen (1994) also presents the integration of humans into HMS, who can enter or exit a holon like other resources, though such ideas can be difficult to implement technically.

One of the earliest and well-known architectures describing holonic production systems is the reference architecture PROSA (Van Brussel et al., 1998). It aimed to provide production systems with greater flexibility and reconfigurability with the vision of creating an *operating system for a factory* (Valckenaers, 2020). ADACOR—an agile and adaptive holonic architecture for manufacturing control—can be treated as PROSA sibling and is PROSA compliant. It provides a multi-layer approach for distributed production and balance between centralized and decentralized structures to combine global production optimization with flexible responses to disturbances (Leitão and Restivo, 2006). Although, many holonic architectures have been proposed, they are mostly variations of the main patterns, described in PROSA and ADACOR. These two architectures also influenced many of our design decisions.

The concepts of agents and holons are very close. As Giret put it in Giret and Botti (2004), “a holon is a special type of agent, and the technology which is used by most people who are dedicated to holonic systems research is the MAS”. The MAS4AI project treats a holon as a special type of agent as well. Agents together with production modules build so-called *resource holons*. Agents associated with products and their models are called *product holons*. How agents together with humans can create *Human Digital Holons* will be shown in the following sections.

Agents, as well as holons, fit very well to designing reconfigurable manufacturing systems thanks to their inherent modularity, intelligence and cooperative capabilities. Several questions must be addressed, though, e.g., how can agents find each other to form a particular configuration, and how do they efficiently coordinate each other to reliably complete a production task? A common approach for tracking agents and their functionalities is to use a so-called Directory Facilitator, as defined in FIPA specification.^1^ For example, a Yellow Pages Agent from Ribeiro and Barata (2013) keeps track of the services each agent in the system offers, so that others could locate it and its services. The information and the services provided by Yellow Pages are not standardized, which hinders the system's interoperability. We use the AAS standard model to provide the discovery functionality for the agents. To form the production configuration, one popular approach is to use some auction-based distributed scheduling protocol, e.g., as in Bussmann and Schild (2001), Knabe et al. (2002), Leitão and Restivo (2006), and Jungbluth et al. (2022). Another, bio-inspired planning ahead approach of the Delegate-MAS (DMAS) from Valckenaers (2019), uses lightweight agents, called ants, for looking ahead and evaluating different production possibilities before the actual resource allocation happens. These elaborate algorithms focus more on flexible production scheduling, leaving the execution to the resources. Though, a real production task is an interplay of many sensors and actuators, which must be perfectly synchronized. That is why, the resource allocation is only one part of the assembly task in our approach, as it will be shown in Section 5.3. Leitão et al. (2003) propose to use Petri Nets (PN) to formally model the behavior of each holon. This enables analysis and formal validation of holon's behaviors. Though the PN is a well-established formalism for modeling discrete event systems, it has some weak points when it comes to the composition of the agents' behaviors. The composed PN model becomes quickly very complex and incomprehensible by humans. In our solution, we use the formalism called Behavior Trees (BTs) which has some nice properties of improved modularity, hierarchical structure and reactivity (Colledanchise and Ögren, 2018). The BTs have been designed to be intuitive for humans and are very expressive.

### 2.2. Digital twins, cyber-physical systems, and Holons

As Ribeiro points out in Ribeiro (2020) there are two emerging trends in designing smart factories, namely Cyber-Physical Systems (CPSs) and Digital Twins (DTs). While Cyber-Physical Systems (CPSs) emphasize a tight intertwinement of a system's computational, physical, and logical parts, DTs are virtual models of physical world objects. Another distinct difference lies in the focus of systems design. CPSs focus on interdependencies and interconnections between physical and cyber, and between different possible autonomous CPSs to collectively create complex behavior. DTs focus more on the synchronization of the real world and its digital model. A CPS autonomously controls the world, while in the case of DTs equipment gets the relevant instructions from its DT (Ribeiro, 2020). The concept of holon can contribute to both approaches. As it was noted in Section 2.1, a holon always consists of an information processing part and, in the case of a resource holon, a physical part. Hence, resource holons fit very well for the implementation of CPSs. Application of CPSs into the manufacturing domain manifests in the concept of Cyber-Physical Production Modules (CPPMs) as the architectural components of the flexible and modular production environments, which provide standardized interfaces to offer different functionalities as services (Kolberg et al., 2018). CPPMs combine with other CPSs and humans to build Cyber-Physical Production Systems (CPPSs).

The concept of AAS as a standardized asset description is widely recognized as a type of DT in the context of Industry 4.0 (Wagner et al., 2017). An AAS consists of several submodels, in which all the information and functionalities of a given asset (features, characteristics, properties, status, parameters, measurement data and capabilities) are described. The AAS serves as the link between the assets and the connected, digital and distributed world, as described by the Platform I4.0.^2^ The AAS provides the assets with the self-description capabilities through the common information model, as well as the standard API to interact with this model. Vogel-Heuser et al. (2021) use the AAS for the initialization of the agents in MAS. In our framework, the AAS plays one of the major roles. We use it to describe every component of CPPS, as well as MAS. We also use the AAS and its API to describe and get access to the parametrization and management services of our MAS. This increases interoperability of our solution through standardization.

A role-based approach for integrating workers into the Industry 4.0-compliant environments by using the AAS to describe the role-related information requirements is presented by Birtel et al. (2019). We extend this approach in our framework by using additionally other worker's characteristics, such as qualification, skills, performance, for better integration of humans into manufacturing environments.

Similar to the AAS model, the modeling approach taken by the Semantic Web and its Resource Description Framework (RDF) from RDF Working Group (2014) should help solve interoperability issues. Although their goals are similar, both the AAS and RDF-based models have their specific strengths. Whereas AAS models are easier to integrate with operational technologies in a production environment, RDF-based models offer more semantic expressiveness for modeling the smart worker and advanced querying. Different approaches have been proposed to bridge the gap between both modeling paradigms. Grangel-Gonzalez et al. (2016) create an RDF representation of an AAS (Semantic AAS) and Bader and Maleshkova (2019) map an AAS model onto an RDF-based schema. In our framework, we propose a method on how AAS models can be created from RDF-based schemas (Rongen et al., 2023). That allows us to reuse the large amount of already existing semantic models in RDF and reference to their URIs directly. We also focus on using both AAS and RDF models in parallel to combine the benefits of both metamodels, by reusing data and functionalities offered by the other metamodel.

### 2.3. Capabilities and skills for the flexible production

A Service-Oriented Architecture (SOA) paradigm as a way of distributed systems is being progressively adopted in the industrial automation domain. SOA is based on the idea of providing and requesting services. A service is a piece of software that encapsulates the functionality of some entity and provides it through the well-defined interface (Leitao and Karnouskos, 2015). Service provider and requestor do not need to know each other, but only the service description. This enables distributed and loosely coupled architectures. Jammes et al. (2007) proposed to use web services for the embedded devices. The project SOCRADES (Cannata et al., 2008) followed these ideas and applied SOA principals in the industrial automation domain. The projects that followed continued to develop the pattern of using adapters to integrate non-service low-level logic of devices to some service-oriented middleware.

The SOA paradigm has found its extension in the industrial automation domain as a skill-based approach, as Dorofeev (2020) calls a skill “a control-level service”. The skill-based engineering paradigm is closely related to a so-called product, process, resource model (PPR), which was introduced to enhance the separation of concern between products, processes, and resources. Motsch et al. (2021) show that the PPR-model enables to focus separately on the capabilities needed to produce a product and those provided by the resources, which are currently available in production. This allows the matching of the required and available capabilities by their functional descriptions and the creation of a production plan that can be dynamically executed by the resources providing the required skills. The latest extension of the PPR model is called a capability-skill-service model (CSS) and was presented by the Platform Industry 4.0 in Köcher et al. (2023). It defines a capability as “an implementation-independent specification of a function in industrial production to achieve an effect in the physical or virtual world”; a skill is “an executable implementation of an encapsulated (automation) function specified by a capability”. We have applied the concepts of capabilities and skills also to all of our holons. This enables a user to find the required holons based on their capabilities. As we describe later, it is also possible to quickly change the implementation of the holon's capability by swapping the corresponding skills.

Ribeiro and Barata (2013) focus on the rapid device deployment for the plug-and-produce scenarios. Their Mechatronic Agent, as the lowest abstraction entity in the framework, directly interacts with the controllers and ensures synchronization between the agent platform and the low-level execution code. The authors admit some shortcomings of their approach, such as, heavy reliance on Java technology and the performance issues that may hinder the system's scalability. In our framework, the lowest abstraction entity is the atomic skill, which is implemented using the OPC UA technology, as in Dorofeev and Zoitl (2018), Zimmermann et al. (2019), and Volkmann et al. (2021). This is an application of the SOA-based paradigm and is aligned with the concept of CPPMs by providing their functionalities as the standardized skills. The producers of the equipment have no need to implement agents or some “harmonization” libraries, instead they focus on the implementation of the standardized skill interface using the OPC UA, which is a de facto standard in the industrial automation world. Thus, we combine skill-based/SOA and MAS approaches. On the execution level, CPPMs provide standardized OPC UA skills. Agents create the intelligence layer and together with CPPMs build the Resource Holons, as it was initially proposed in Ruskowski et al. (2020).

### 2.4. Human-centered cyber-physical systems

The era of computer-integrated manufacturing (CIM) of the 1980s, with the vision of fully automated plants that would make human workers obsolete, has proved the very opposite: the need to build manufacturing around and for humans (Zuehlke, 2010). Another, so-called “techno-centered” approach, places a human in charge of making all the decisions. The “magic human” in Trentesaux and Millot (2016) is assumed to solve all the problems that may appear during manufacturing. The authors provide several examples that highlight the overestimation of such assumptions. They also propose some recommendations for designing more “human-centered” manufacturing systems:

- Humans must always be aware of the situation;
- Repetitive actions/decisions must be avoided;
- Rare situations, for which an operator is not well-prepared, must be cleared as soon and as much as possible;
- The human mental workload must be carefully regulated to avoid mental overload, stress, fatigue, or boredom;
- The level of system automation must adapt to situation and human competence.

Such agents would succeed in the situations where neither human agents nor machine agents can do alone. Peruzzini et al. (2020) stress the importance of human-centered design and human factors for human-machine interactions in Operator 4.0. Sparrow et al. (2022) present an architecture to facilitate the integration of human workers into Industry4.0 environments. Their solution combines the AAS, as described by the RAMI4.0 (DIN SPEC 91345, 2016), the holonic architecture in accordance with the ARTI design principles (Valckenaers, 2019) and the concepts from Operator 4.0 (Romero et al., 2016). Conceptually, this work is the closest to our approach. The authors focus on the implementation of the AAS for a human worker, but it is not clear, if they follow the set of specifications, defined by the platform Industry 4.0.^3^ In our solution, standardization plays the major role in ensuring interoperability between the system's components. We rely on the standard AAS metamodel and the set of APIs, defined in the specifications. The focus is made on the semantic modeling aspects for the AAS and its combination with the holons. The worker's AAS is seen as the human's passive digital twin, while the holon actively represents the human in the system. Furthermore, the skill-based concept allows designing the main elements and interfaces in an implementation-neutral way.

To sum up, the main objective of this work is to investigate how the methodology developed in our project for integration of various AI technologies into distributed production environments can be applied to improve the interaction between humans and machines. By realizing the vision of Operator 4.0 we benefit from the complementarity of humans with AI technologies for solving problems that seemed unattainable before.

## 3. Key concepts for human-centered smart manufacturing

The goal of the MAS4AI project is to develop and test a distributed and interoperable architecture based on AI multi-agents technology. AI-powered production systems in cooperation with humans can improve planning and execution and help to increase the quality of products and processes. This will contribute to the hyper-agility of European factories through human-assisted autonomous, modular, and reconfigurable production while at the same time keeping humans in control of AI technology.

As discussed in Section 1 on of the main challenges toward effective smart manufacturing is to integrate different state-of-the-art AI technologies with advanced manufacturing control, respecting safety, security and ethical aspects. To face this challenge, we employ a holonic multi-agent system, based on the RAMI4.0 reference architectural model and the use of AAS. The AAS is envisioned to facilitate the digital representation of all the components in the system: production resources, agents, AI-algorithms and smart workers. This enables monitoring and information exchange in a unified and interoperable manner.

**Figure 1:**
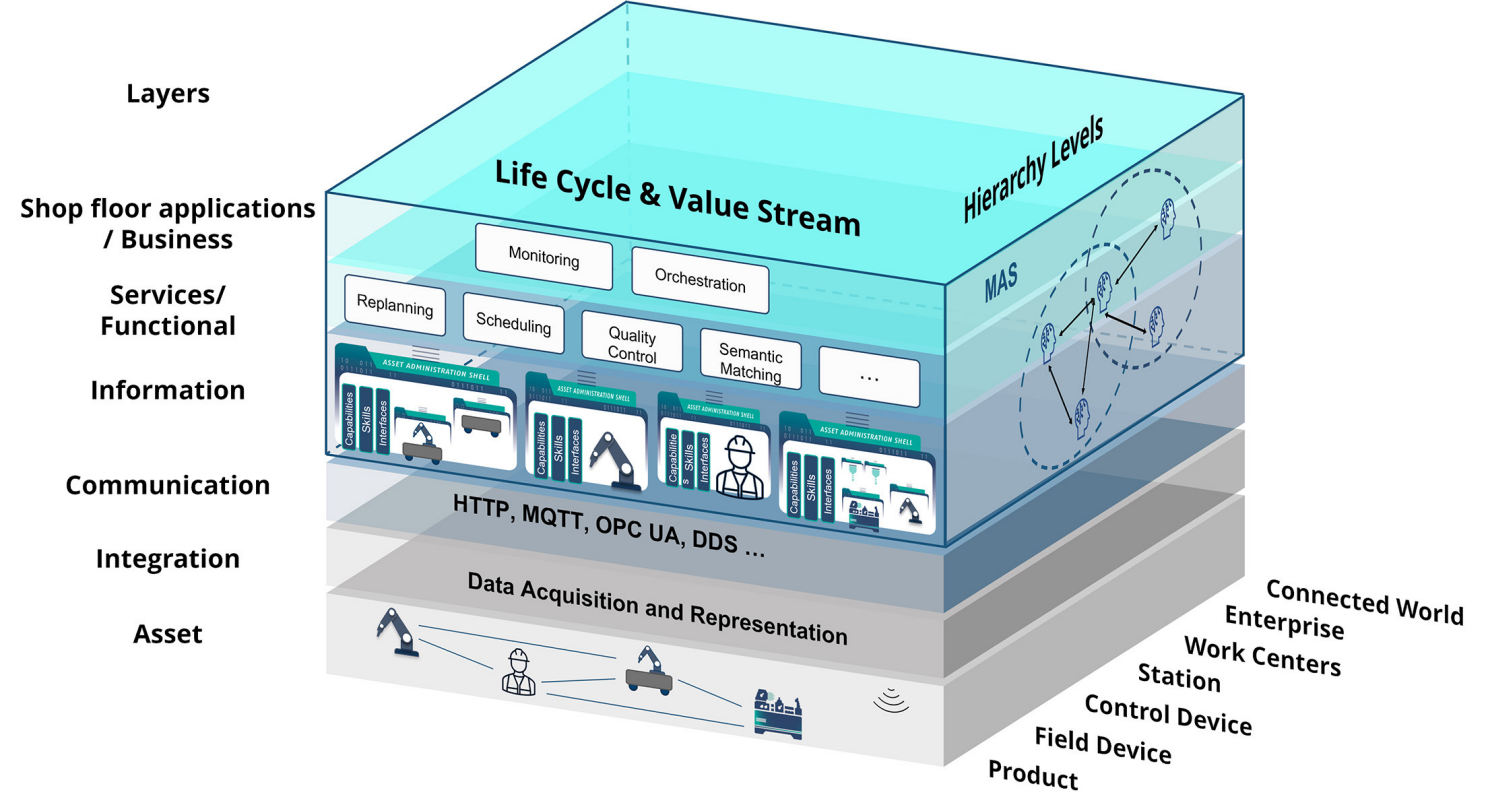
Alignment of the MAS4AI Solution with the RAMI4.0 Architecture (based on DIN SPEC 91345, 2016; Alexopoulos et al., 2020; Popper et al., 2021).

Figure 1 shows how the MAS4AI solution aligns with the RAMI4.0 reference architecture model. MAS4AI agents and holons find themselves on the three upper layers of the “Layers” architecture axis of the model. On the information layer, all the assets are uniformly described by their AASs to enhance interoperability. On the one hand, agents utilize the structure of the AAS to autonomously get the required information, on the other, they are also described by their AASs in a standardized way. AI algorithms may be part of agents, but can also be provided as services of the functional layer. Agents can both provide and use these services. The business layer is driven by different business use cases, e.g., monitoring or production orchestration, where agents and holons take active part. MAS4AI agents are present on all the levels of the “Hierarchy” axis of the RAMI4.0 model. Product holons ensure proper production of products, while resource holons represent production resources, e.g., devices, stations or work centers. Holons of the Enterprise and Connected World levels take part in the Shared Production scenarios.^4^ The “Life cycle & value stream” axis is also represented in the MAS4AI solution through type and instance AASs of assets and agents (not shown in the figure).

Finding effective ways for collaboration between humans and AI systems, and exploiting the strengths of both humans and machines while keeping the human in control, is one of the core research topics of the project. For better integration of humans, whom we call smart workers, into production systems, we use key concepts that are described in the following sections. Section 3.1 discusses human's semantic models to describe a smart worker precisely and unambiguously. The smart worker's AAS, described in Section 3.2, enables the standardized and secure access to all the information a system needs about a worker. In Section 3.3, we clarify the concept of the Human Digital Holon for the symbiotic integration of humans into holonic multi-agent systems.

### 3.1. Semantic models of a smart worker

Formal semantics offer a precise and unambiguous way of representing information regarding the human worker. They allow for automated interpretation by machines and reasoning over knowledge about the worker. Semantic models of the smart worker aim to capture concepts that allow the human to effectively collaborate with other agents and machines. The model of the smart worker contains descriptions of concepts, all made available via the digital persona (representations) of the human. These include:

- Skill model: representing the technical knowledge and skills possessed by the worker. This includes information about the worker's education, training, and work experience, as well as specific competencies and certifications they have earned. Standards that express concepts for this model include ESCO^5^ and O*NET.^6^
- Qualification model: representing qualifications required for the worker to perform their job, such as professional licenses or degrees. It could also include information about the worker's language proficiency and other soft skills that are necessary for effective communication and collaboration.
- Personality model: representing the affective state of the worker, including their personality traits, motivation levels, and stress tolerance. It could be used to predict how the worker might respond to different types of work environments or situations.
- Performance model: representing the worker's performance metrics, such as productivity, quality, and safety. It could be used to identify areas for improvement and to monitor the worker's progress over time.
- Context model: representing the context in which the worker is operating, including the specific task they are performing, the equipment and/or resources they are using, and the environment in which they are working. It could be used to optimize the work environment for maximum efficiency and safety.
- Cognitive model: representing cognitive abilities of the worker, such as attention, memory, and perception. It could be used to design tasks and training programs that are tailored to the worker's cognitive strengths and weaknesses.

**Figure 2:**
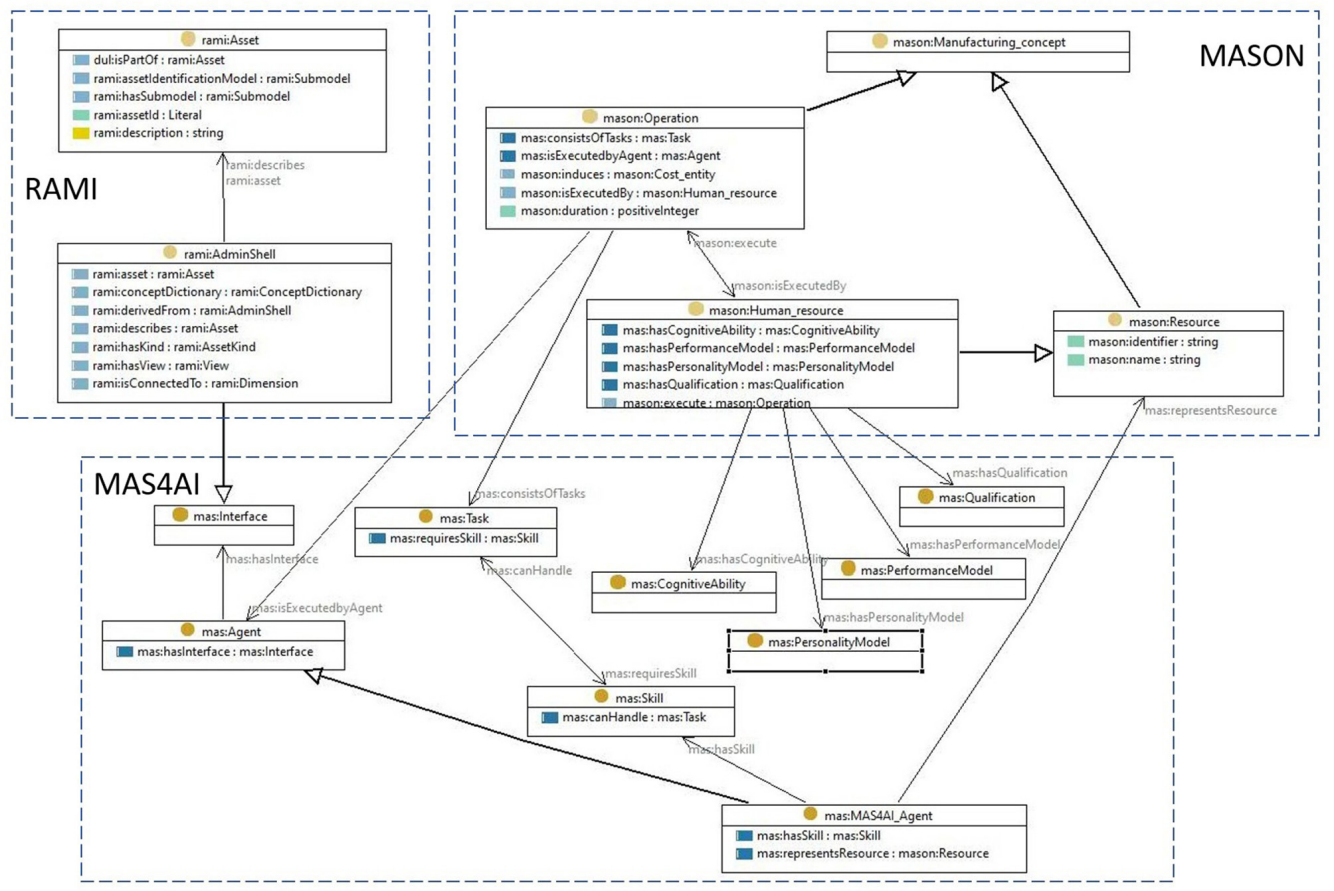
High-level ontology for a smart worker.

An ontology for the smart worker that includes the concepts of the above can be defined by extending ontologies available in the manufacturing domain in order to reuse existing and well-founded knowledge models. Figure 2 shows the main concepts of the smart worker and relations between concepts defined in such an ontology. MASON (Lemaignan et al., 2006) provides a core model that conceptualizes core concepts of the PPR model (Cao et al., 2019), defined as Entities, *Operations* and *Resources*. A MAS4AI ontology for the smart worker then implements the notions of an *Agent* as a specific representation of Resources, it adds *Task* to describe the context of the worker, as well as *Skill* required to execute the tasks, the *Qualification* models, models of the Affective state (*AffectiveStateModel*), *PerformanceModel* that registers performance metrics and the representation of the *CognitiveAbility* of a human worker. The RAMI Ontology^7^ represents the Reference Architecture Model for Industry 4.0 (RAMI), including the concept of an Administration Shell I4.0 Component (*AdminShell*) for a smart worker.

Describing these models as reusable components (submodels) within the framework of the AAS, allows the creation of new open models of the smart worker which are interoperable with already existing models and fit the MAS4AI framework.

### 3.2. Asset Administration Shell for smart worker

In the proposed framework, each asset, e.g., a CPPM, a smart worker or an agent, is described in a standardized way by its AAS. The MAS4AI agents use the standard AAS API to get all the required information about the assets. In this section, two types of AAS are described—a smart worker AAS and an agent AAS.

**Figure 3:**
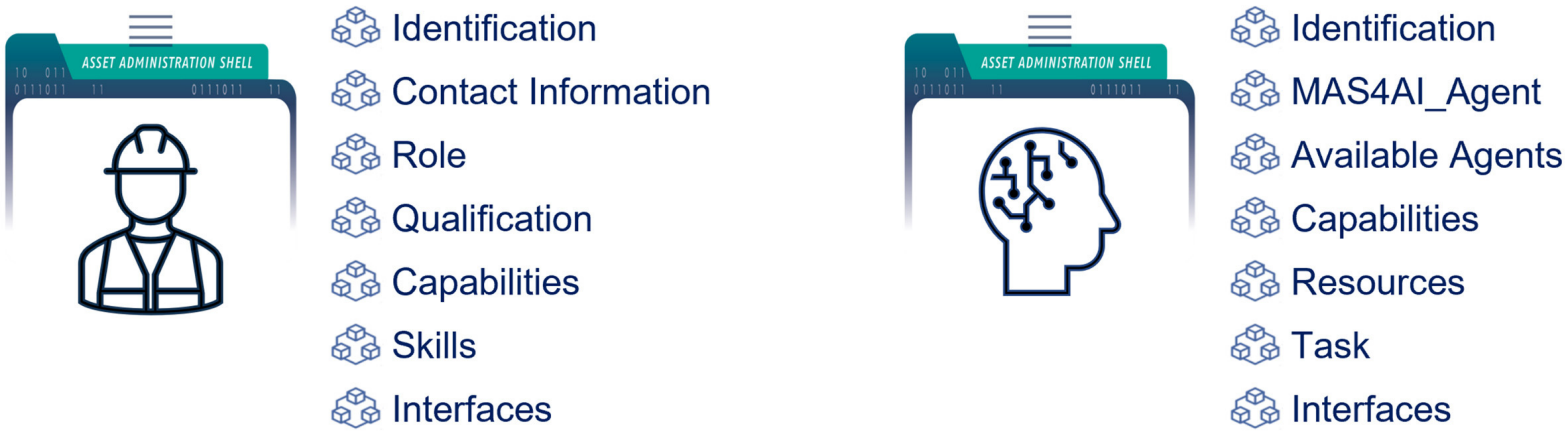
AASs for a smart worker and a MAS4AI agent.

Figure 3 shows the main submodels of the smart worker AAS. Some submodels, such as *Identification* are common for all the assets, others are unique for specific AAS types. The smart worker AAS provides information about a worker's role, qualifications, capabilities, and skills. Some semantic models described in Section 3.1 have not yet been included in the current version of the smart worker AAS and need more elaboration and standardization. The *Role* submodel describes which role a person has in the organization. The system uses the role information for the proper interaction with the human. For example, different roles imply different access levels or different agents associated with the person. The worker's role in the production can change depending on his/her location or task. The system can monitor both and accordingly update the worker's capabilities and performance. Moreover, due to the worker's physical conditions, e.g., fatigue, his/her capabilities and performance can also deteriorate. This can lead not only to quality issues, but also to potentially dangerous situations and accidents. To prevent such situations, the system must carefully monitor the worker's conditions and promptly update the corresponding AAS submodels, and, if needed, raise an alarm. The *Qualification* and *Skills* submodels provide insight into the worker's ability to perform a certain task and can hold the results of performance monitoring/evaluation and various tests. The *Capabilities* and *skills* submodels are common for the resource assets and are used to realize the PPR model as described in Section 2.3. The *Interfaces* submodel lists the interfaces, which are currently available to interact with the smart worker, e.g., cameras, tables, AR-glasses, etc. This information is also sensitive to the worker's location. As it will be shown later, these interfaces are normally not just devices, but so-called *Human Interface Holons*.

Figure 3 also shows the AAS for the MAS4AI agent. The *MAS4AI_Agent* submodel is used to describe a particular type of the MAS4AI agent and has a reference to the agent's semantic model. It also indicates if the agent has holonic properties, i.e., consists of the other agents. If an agent is a holon, then the *Available Agents* submodel lists the subordinate agents. The *Resources* submodel shows all the resources available to the holon, e.g., production resources or human resources. The *Task* submodel describes the production tasks that are currently running or scheduled in the holons agenda. The *Interfaces* submodel describes all the interfaces available to the agent. It includes the communication channels, e.g., MQTT, OPC UA, or REST, as well as interaction protocols, e.g., the FIPA ACL^8^ or the I4.0 language (VDI/VDE-GMA, 2020a).

In the proposed framework, both RDF-based and AAS models are used in parallel to reinforce each other. RDF is used to build powerful descriptive models, whereas AAS provides standard structure, interface, and better integration with the physical world. The semantic model shown in Figure 2 together with the SPARQL technology^9^ can be used to improve the discoverability of assets in the distributed MAS environment. Each holon and asset in the framework is represented by its AAS, which provides the connection to the physical or virtual entity. Though, to find the holon with the exact required capability can be a non-trivial task because the AAS lacks the querying functionalities, as provided by the RDF framework. Rongen et al. (2023) describe a method how one can use the RDF store and the dedicated knowledge graph for creating a query to find the required AAS for further use by utilizing the standard AAS's mechanisms. In the context of the Human Digital Holon, which will be described in the next section, the agent's AAS serves two main purposes: to describe the holon in a standardized way and to interface with the smart worker context as described in Section 3.1. The knowledge graphs, based on the model shown in Figure 2, enable the holons to reason about the smart worker and to make informed decisions.

### 3.3. A Human Digital Holon

As discussed in Section 2.4 the need for better integration of humans in the industrial environment in sense of human-machine symbiosis has been identified by many authors (Romero et al., 2016; Trentesaux and Millot, 2016; Peruzzini et al., 2020). While personal digital assistants play a subordinate role to humans by executing human requests, a vision of Human Digital Holons lies in a more symbiotic relationship between human agents and artificial agents and in the creation of hybrid agents. Such agents will succeed where neither of the former ones can produce sufficiently good results (Romero et al., 2016).

Sousa et al. (2007) note that HMSs can “effectively integrate human operators in the manufacturing process” and will require bidirectional Person-Machine Interfaces. On the other hand, Valckenaers (2019) argues that humans lack presence in the digital world and thus cannot effectively communicate with other holons. Sparrow et al. (2022) also point out that though humans are a good example of holons, they cannot be easily integrated with digital holons and need some extensions. According to them, there are tree main responsibilities of the human digital holon:

- To represent a human in holarchy and to interact with other holons on a human's behalf;
- To provide a human with only necessary information to avoid cognitive overload;
- To establish an efficient interface between a human and a system.

**Figure 4:**
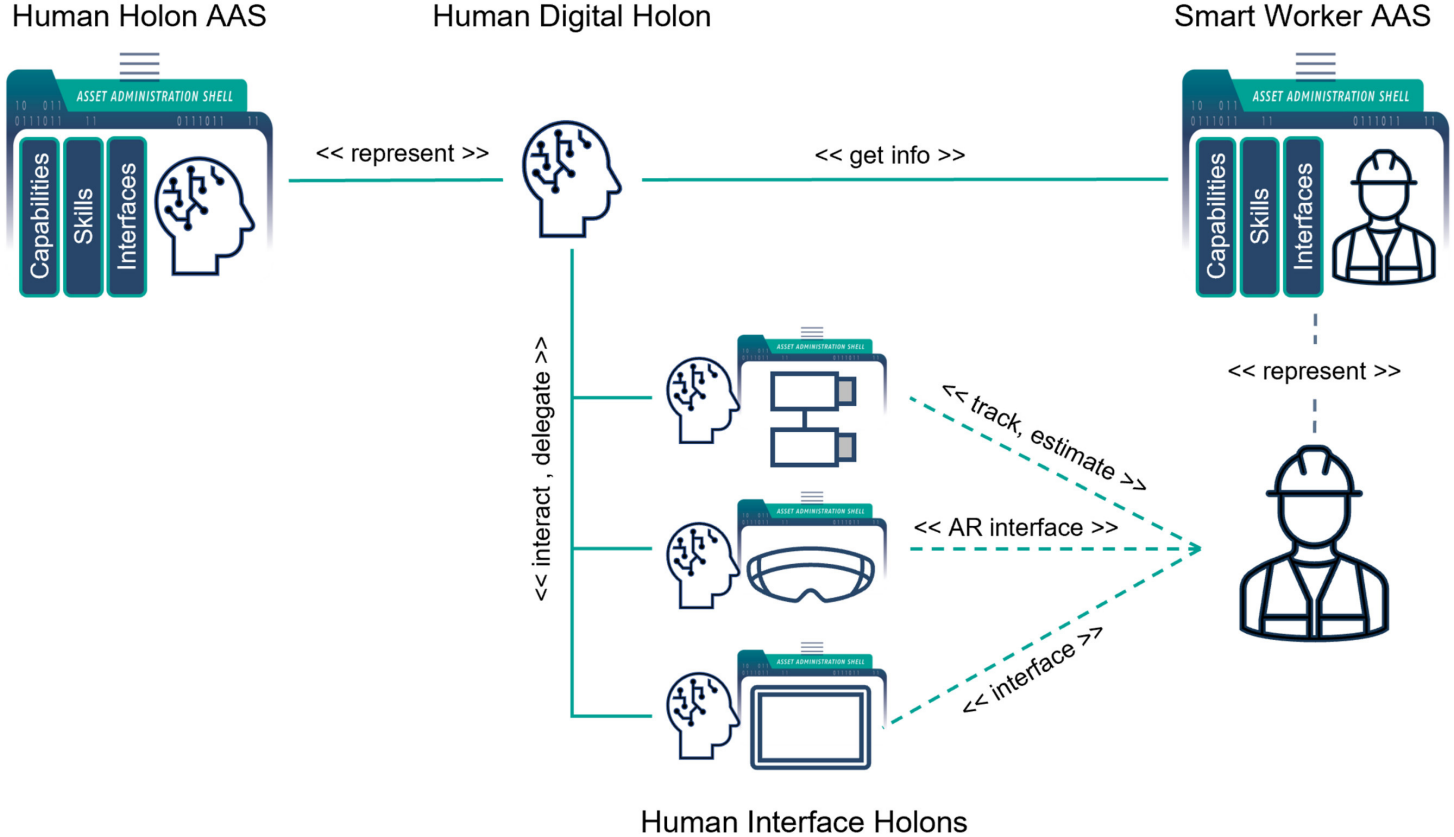
Human Digital Holon.

Our approach is to combine the worker's AAS as human's passive DT with the Human Digital Holon, which actively represents a human in the system. To augment humans with necessary information and build with them the efficient interfaces, the specialized holons are used, called *Human Interface Holons*. Figure 4 shows a high-level structure of the human digital holon. To interact with the other holons on behalf of the smart worker, the Human Digital Holon needs necessary information about a person. The human AAS described in Section 3.2 serves as the single source of truth about the worker and is a part of the Human Digital Holon. The holon uses the standard structure of the AAS submodels to take part in the negotiations, e.g., in the contract-net-protocol (CNP) (VDI/VDE-GMA, 2020b) for the resource allocation. It also updates the AAS depending on the actual situation. For example, the role of the worker in the production process may change depending on the person's location. The holon tracks the worker's location and updates the role in the AAS. Depending on the worker's qualification and role, the type of the holon can also differ, e.g., an assembly module operator is represented by a Resource Holon, whereas a process expert can take part in planning or optimization activities as a Supervisor Holon.

To provide humans with only necessary information and support them in making decisions, the specialized holons inside the Human Digital Holon, the Human Interface Holons, get data from the smart sensors, analyze the current situation and the production goal and make the worker to selectively perceive the information that is important in the current moment to make quality decision. In general, it is difficult to integrate human beings and digital holons by directly connecting them. The traditional means such as graphical user interfaces (GUI) or mechanical buttons may not be enough for sufficient integration of humans and holons. The task of the Human Interface Holons is to indirectly interface with humans by using AI algorithms and data from smart sensors.

## 4. Proposed architecture

### 4.1. Overview

**Figure 5:**
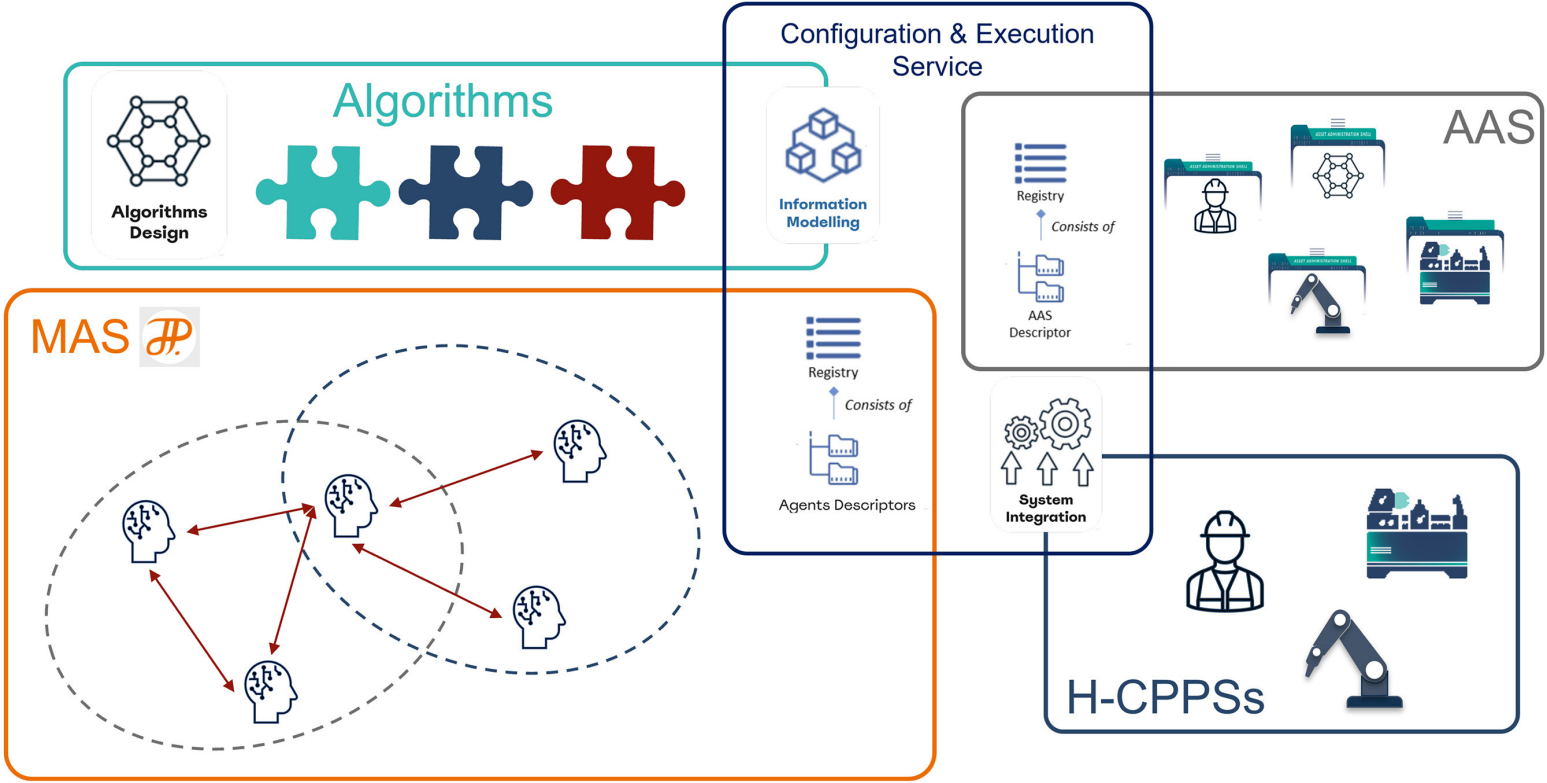
MAS4AI framework overview.

The proposed framework as described in Sidorenko et al. (2023) consists of several components that are combined to build a holonic architecture to facilitate the integration of AI technologies, CPPMs and humans with the concept of the AAS. An overview of the framework is illustrated in Figure 5. The AAS-hosting platform provides the AASs for all the assets including CPPMs, humans, agents, as well as algorithms. The self-description capability of the AAS is used for the system's configuration and parametrization. All the components of the framework can be exchanged if they follow the general architecture of how the components and their interfaces are structured and described. The MAS used in the project is based on the Janus runtime for the general-purpose agent-oriented programming language SARL (Galland et al., 2020). The decision in favor of SARL has been made because of its explicit support of holonic architectures. SARL also provides useful abstractions, such as capacities, skills, and behaviors, to model agents' functionalities. These constructs follow the skill-oriented approach, which was discussed in Section 2.3, and ease the agents' development, as it was evaluated in the prototypical testbed environment of the SmartFactory-KL (Motsch et al., 2023).

The MAS4AI framework provides the templates for the commonly used holons, as well as their standard description in the form of the AAS. This allows to quickly deploy the required holon types for different applications, for example, resource control, monitoring or production planning, as well as to realize complex holonic control structures, which can consist of several MAS runtimes, each representing the subset of the system's holons.

The integration of different components into the MAS is based on the standard AAS model, as well as on the infrastructure and the APIs defined by the AAS standard. The MAS4AI holons implement the AAS REST API to interact with the AAS-hosting platform and use the standardized structure of the AAS submodels to describe themselves, as well as to get the required information about others. The AAS model is also used as a facade to the configuration and execution services of the MAS4AI framework. This ensures independence from the implementation technologies and conformance to the Industry 4.0 standards. Two AAS-hosting platforms, BaSyx^10^ and Dimofac,^11^ have been used in the framework, and the MAS4AI agents could work with both of them by using the proper skills. This shows the extensibility of the framework and its ability to work with different AAS technologies.

### 4.2. Building a Holon in the MAS4AI

This section describes how the holons are built in the MAS4AI framework, as well as different ways of integrating AI-algorithms as the holons' programs.

**Figure 6:**
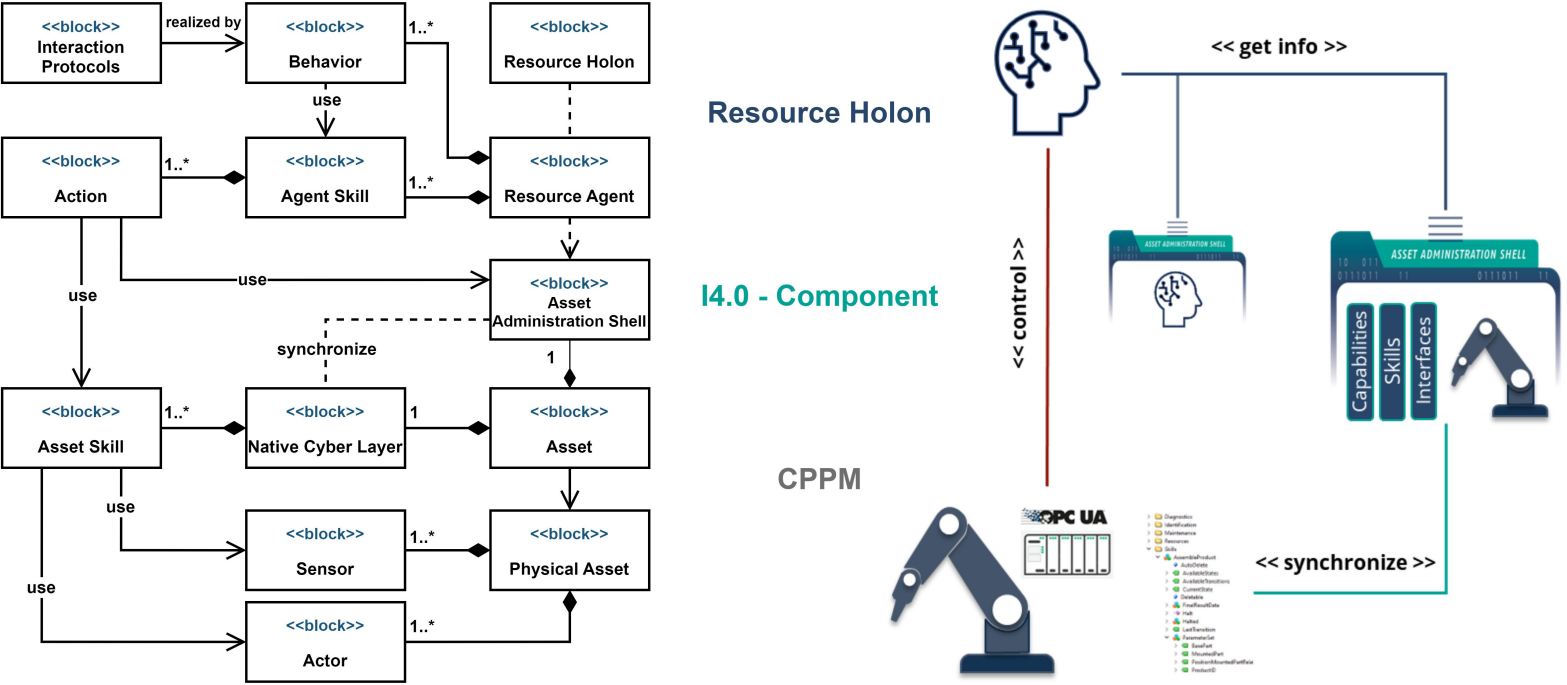
Resource Holon. The asterisk ^*^ denotes unlimited number of elements in the upper bound of the UML multiplicity.

A Resource Holon usually represents some production resource, for example, a production module, transportation vehicle or a smart worker. Figure 6 shows a block diagram of the resource holon, which represents a CPPM. The CPPM typically consists of a set of actuators and sensors, which interact with the environment to complete some task. It also has its own computation platform and a software layer, or a native cyber layer, which controls its hardware and provides the interfaces for the external users. We follow the skill-based approach for designing CPPM's control architecture and encapsulate the module's functionalities as skills with standard interface and behavior. For example, for the robot in Figure 6 such a skill can be “*pick and place”* and is provided as an OPC UA skill model presented in Volkmann et al. (2021). To be a part of the I4.0 ecosystem, the CPPM needs to have the standard digital representation in the form of the AAS. This makes the module a so called I4.0 component. The module's AAS synchronizes with its native cyber layer to provide the module's status and services to the other I4.0 components. Some of the most important AAS submodels used in the project are *Capabilities* and *Skills* to follow the CSS model described in Section 2.3, as well as *assetInterfaceDescription*,^12^ which provides the description of the asset's native interfaces to enable the automatic configuration of the communication channels. Agents build the intelligent layer of the CPPM. The SARL agents also have skills as part of their model. A SARL skill consists of actions, which implement some algorithms or call external services. In the Figure 6, the agent's skill has a sequence of actions to use the underlying module's skill. The actions use the information from the AAS submodels to connect, configure and properly use the skill. The SARL agent also has a set of behaviors, which consist of event handlers and event emitters. This enables to implement different interaction protocols, e.g., contract-net-protocol (CNP), which can be used for dynamic planning and resource allocation (Jungbluth et al., 2022). The resource agent, together with the CPPM and the AAS, builds up the Resource Holon.

**Figure 7:**
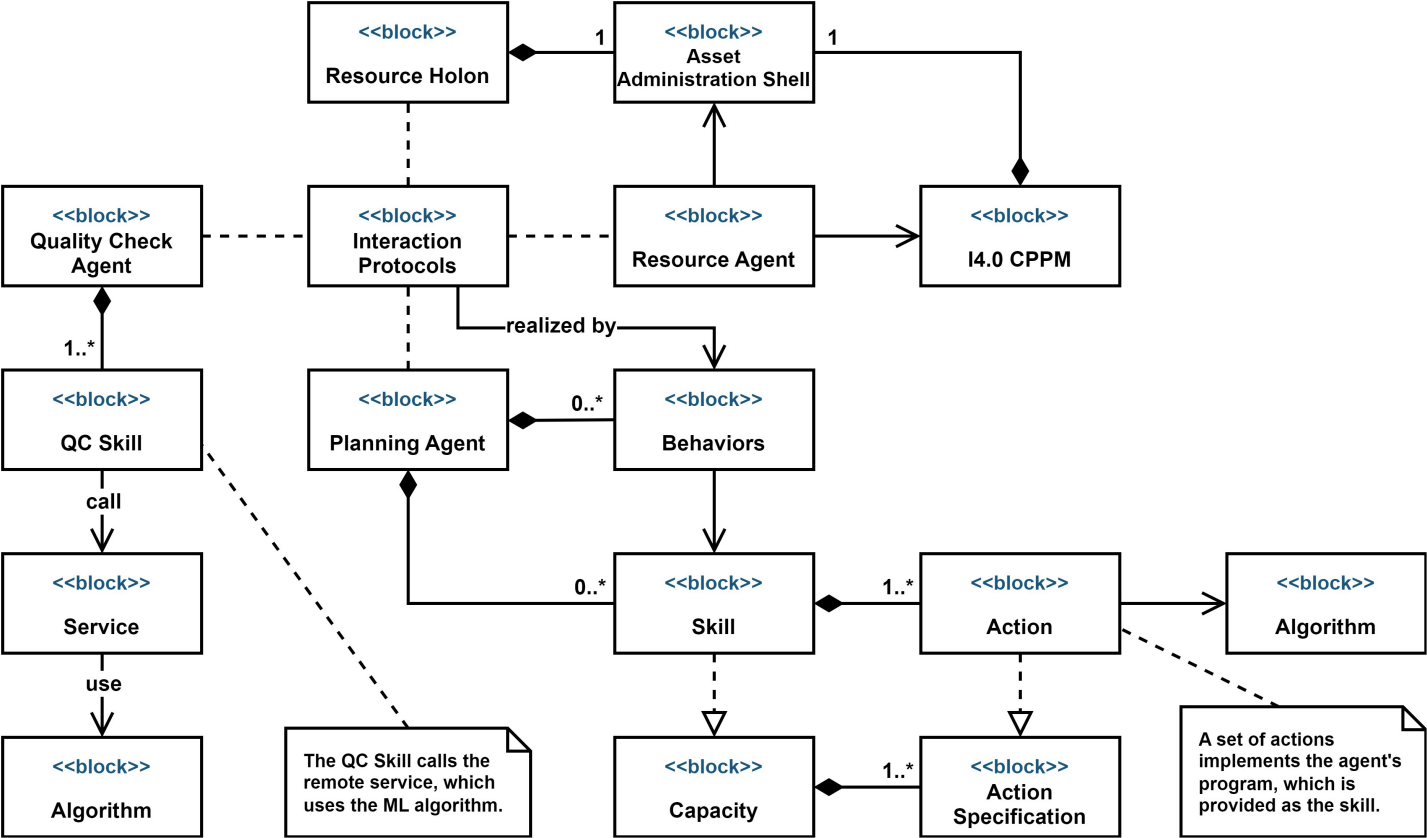
Integration of AI-algorithms with the MAS4AI agents. The asterisk ^*^ denotes unlimited number of elements in the upper bound of the UML multiplicity.

An agent's function describes an algorithm that maps the agent's perceptions to its actions. An agent's program is the implementation of this function, which runs on a specific computational platform. Figure 7 shows the possible integrations of the AI-algorithms as the MAS4AI agents' programs. There are three integration mechanisms that we use:

- The agent's program is implemented and deployed externally, and the agents use it through a set of services;
- The agent's program is implemented using SARL language abstractions and directly integrated into the agent;
- A combination of the two previous variants is also possible.

The first variant assumes that an agent's program is implemented and deployed externally, and is provided as a service. In Figure 7, an exemplary pattern recognition algorithm runs on an edge device and provides its service via REST API. A *quality check agent* has a skill that uses its actions to make direct calls to that service to query the algorithm. This approach separates the algorithm's development from the agent's development. The agent's program implementation can be freely changed as long as the interface stays the same. The skill-based approach can also be used in that case. For example, a functionality of an ML algorithm, which is provided by the pattern recognition system, is implemented as a skill with the standard skill model and the agent knows how to use it. The AI algorithms are also treated as the assets in the MAS4AI and thus are provided with the AAS.

In the second case, the SARL constructs of capacity, skill, and action are used for the implementation of the agent's program. A SARL capacity is the specification of a collection of actions, whereas a skill is the implementation of this capacity. It should be noted that SARL capacity is conceptually very close to the notion of capability, which we used before. We use capacity here to be consistent with the SARL terminology. The actions of the skill can be executed as a reaction to some external events or proactively, i.e., triggered by an internal event. The combination of the actions and the events that trigger these actions builds the actual agent's program. Skills and capacities enable code reuse and modularity. In the Figure 7, a planning agent has a planning capacity that defines an interface for a planning skill. This capacity can be implemented by several skills, each representing a different planning algorithm, as long as the capacity's actions specifications are respected. Thus, the agent can change the algorithm it uses by simply instantiating the proper skill.

### 4.3. Testing the framework

During the project lifetime, several prototypes have been developed for testing different aspects of the proposed framework, such as integration with the CPPMs, interactions between holons and with the external services, scalability, integration of different AI-algorithms and technologies (Motsch et al., 2023). Integration with the CPPMs follows the same principle, as shown in Figure 6. Every CPPM provides its functionalities as the OPC UA skills with the standard interface and behavior. They are exposed by an OPC UA server, either built into a PLC or provided by an OPC UA adapter (*Native Cyber Layer and Asset Skill blocks* in Figure 6). For the interactions inside one runtime, the holons use Janus native event-based mechanism. To increase scalability of the framework, we run different holons in separate runtimes inside Docker containers. For the communication between such holons we use Kafka^13^ to build a robust agent messaging system. The I4.0 language is used as the agent communication language. we have tested our framework on several demonstrators and with the different industrial automation hardware. As it was mentioned in Section 4.1 two different AAS platforms, BaSyx and Dimofac, have been used as the AAS infrastructures. Though, the demonstrators are still only the lab-scale proof-of-concept prototypes and cannot provide the real production data and results.

MAS4AI involves challenging use cases to demonstrate feasibility, scalability, and flexibility of the framework for the deployment of AI solutions in different hierarchical layers of modular production in a wide range of sectors (automotive, wood, bicycles, bearings, and metal). Various AI technologies have been used to create agents for the pilot-lines use-cases, e.g., hierarchical planning, model-based machine learning, reinforcement learning, etc. The algorithms have been implemented using different software frameworks and integrated in the MAS4AI platform following the approach described in Section 4.2. As the project is still running, the pilot-lines are currently in the final stages of deployment phase. In the following verification and validation phase, the framework can be evaluated against the KPIs set at the beginning of the project.

## 5. Case study: a human-robot shared assembly task

### 5.1. Testbed description

The presented testbed demonstrates a reconfigurable CPPM, which is built from a set of exchangeable submodules around a handling central module. Each submodule comes with the standard mechanical, electrical, and software interfaces to ensure the plug-and-produce scenarios. The CPPM can be reconfigured to produce new types of products by introducing new submodules that add new capabilities to the system. There are several requirements for the testbed that must be satisfied:

- The control must be distributed across different control systems of the submodules because the centralized control system approach will not support rapid reconfigurability;
- The submodules must be described by a common information model to ensure interoperability and integration;
- The system must configure itself to enable plug-and-produce scenario;
- The system must ensure the efficient orchestration of the submodules' functionalities to complete various assembly tasks;
- The smart worker must be seamlessly integrated into the system considering his/her role and skills;
- The system must respect the recommendations proposed in Trentesaux and Millot (2016) and listed in Section 2.4.

The control system of the testbed uses the MAS4AI holonic approach and is influenced by the ADACOR architecture (Leitão and Restivo, 2006). The Resource Holons of the MAS4AI are similar to the Operational Holons of the ADACOR and the Product Holons correspond to the Task Holons. The Task Holon of the MAS4AI corresponds to the Supervisor holon of the ADACOR and is also motivated by the need to coordinate different Resource Holons to perform a common task. Each Resource Holon represents a smart device, a production module or a worker. The Resource Holons form together the control structure of the CPPM. It is the responsibility of the Task Holon to coordinate the Resource Holons during production. When new equipment is introduced, it brings new capabilities to the system and the new production tasks can be accomplished.

### 5.2. Human Digital Holon self-configuration

One of the enablers of the plug-and-produce scenarios is the assets' self-description capability. As it was discussed earlier, all the assets in the MAS4AI project use their AASs to provide all the information needed by the system for their proper integration. This section shows how this approach can be used to support self-configuration of the Human Digital Holon, as well as each Resource Holon in the system.

The scenario is shown in Figures 4, **9**. The testbed has a hand-assembly submodule where a worker helps a robot to accomplish tasks that need some dexterous operations, which the robot cannot accomplish by itself. Without the worker with the proper qualification, the required skills are absent in the system and the task cannot be completed.

**Figure 8:**
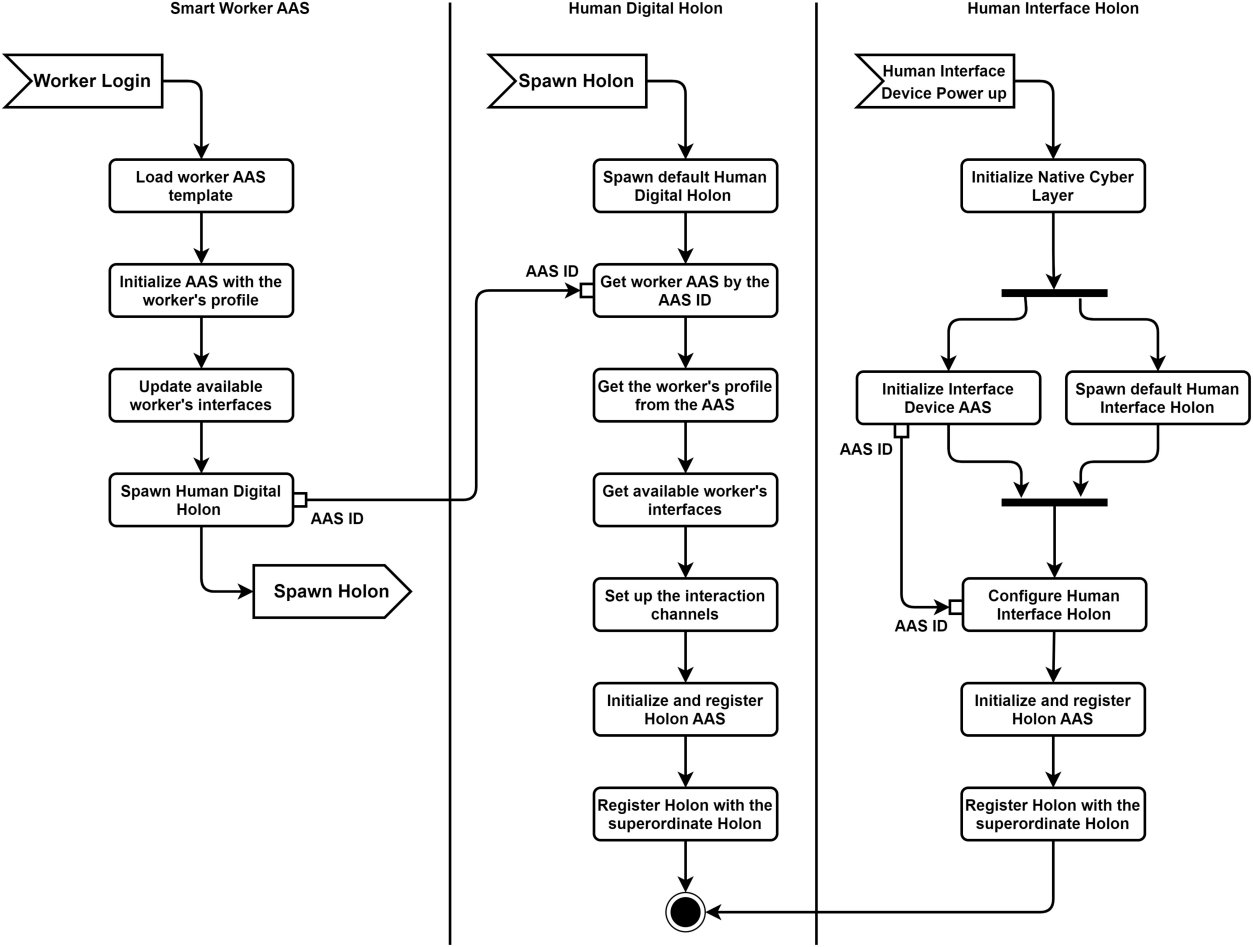
Self-configuration scenario activity diagram.

The simplified activity diagram of holon's self-configuration in provided in Figure 8. When the worker first logs into the system, the corresponding AAS template is loaded to the AAS hosting platform and initialized with the worker's profile (*Smart Worker AAS* part of the diagram in Figure 8). All the information regarding the worker's role, capabilities, skills, as well as interfaces, through which the system can interact with him/her, is filled into the proper submodels. This information is sensitive to the worker's current position.

At the same time, a human interface device, e.g., a smart camera or AR-glasses can be plugged into the system (*Human Interface Holon* part of the diagram in Figure 8). The device initializes its native cyber layer (see Figure 6), its AAS, and spawns the default Human Interface Holon, which configures itself from the device's AAS. The dedicated algorithms needed by the Interface Holon may be provided by the smart device itself as the skills, or run as the external services. In both cases, the information required for the configuration is taken from the corresponding AAS. After the holon is ready, it registers itself with the superordinate holon.

After the worker has logged in and got the AAS, the default Human Digital Holon is spawned with the corresponding worker's AAS ID (*Human Digital Holon* part of the diagram in Figure 8). It finds the worker's AAS by the AAS ID, which it gets as a parameter during its activation, and configures itself with the worker's profile information from the worker's AAS. It also checks for the currently available Human Interface Holons in the superordinate holon by reading its AAS. If there are such holons, it connects to their AASs and gets the required information to set up the interaction channels. The holon now announces itself through its AAS as the worker's Human Digital Holon with the appropriate skills and capabilities further to the system. The system updates its available skills and can decide further whether it can accomplish the task or not.

### 5.3. Human-robot coordination for shared assembly tasks

Though the worker has agency and can coordinate the assembly task, whereas the robot acts as a helper and reacts to the worker's commands and actions, often the human might need the system's support. For example, a novice worker might not know what to do next, especially with the highly customized products. New skills may be required to accomplish tasks needed to produce a new type of product.

On the other hand, the robot might not be capable of reliably predicting the worker's actions and thus safely and efficiently helping the human. In this case, more deterministic coordination of both human's and robot's actions can be beneficial. When collaborating with the robot, the worker also wants to feel safe. For that, the transparency of the process and the understanding of what the system is doing or intended to do is crucial. A process of executing a task can be divided into 3 stages:

- Resources allocation: the resources required for a production task are found and scheduled.
- Task configuration and parametrization: the task is configured for the execution and the skills of the production resources are parameterized.
- Task execution: the task is executed by coordinating the skills of the production resources.

#### 5.3.1. Resources allocation

**Figure 9:**
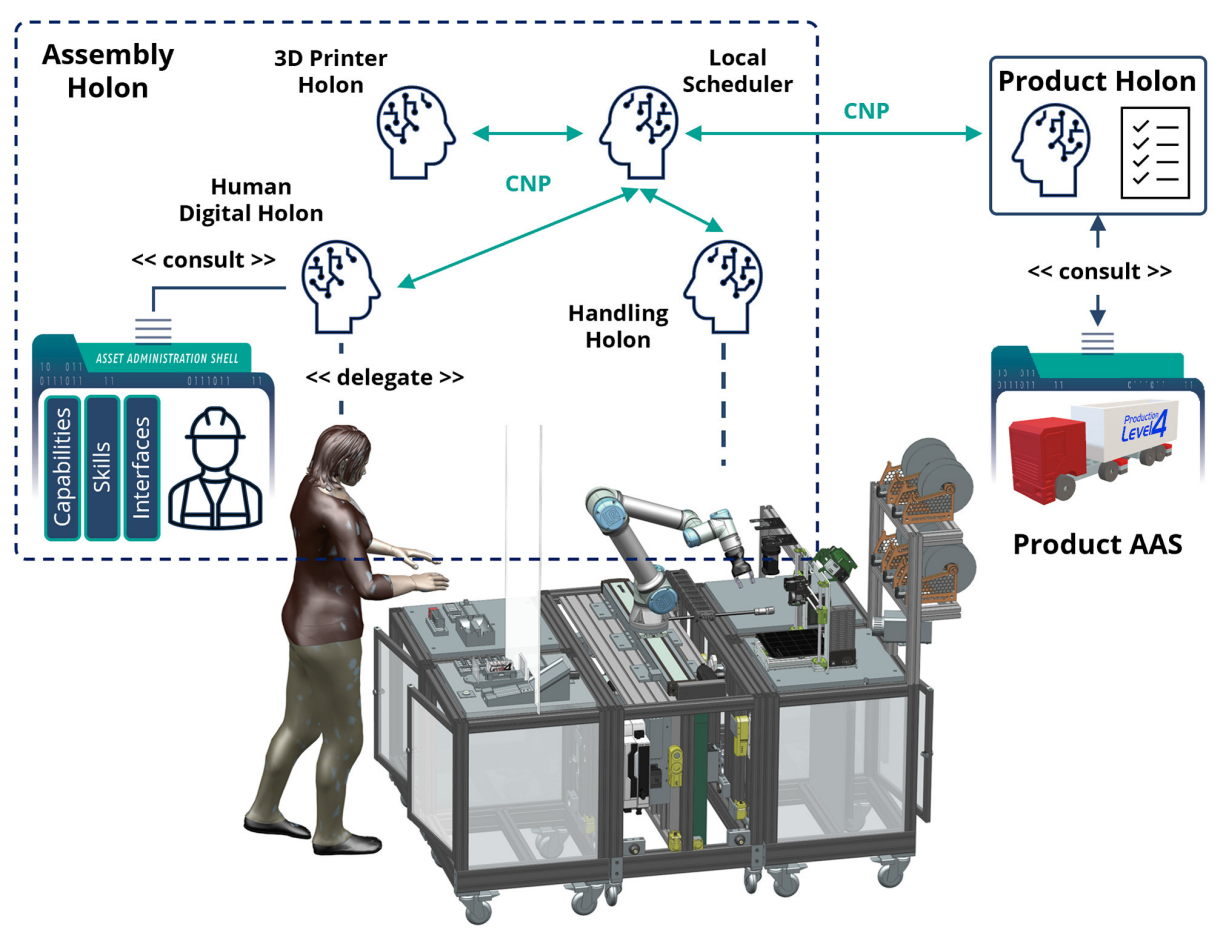
Resources allocation for the shared assembly task.

Figure 9 shows the resource allocation stage of the shared assembly task scenario. A set of capabilities needed to produce a specific product is derived from the product model to match the capabilities of the resources available in the system. In the Figure 9, the product Holon consults with the product AAS on which production steps must be performed next. A step is formulated as a set of capabilities with the constraints, which must be met to carry out this step. The task of the Product Holon is to find and reserve the production resources that provide such capabilities. In this scenario, the agents use a well-known contract-net-protocol for the negotiation and allocation of the resources required to complete a production task. The Human Digital Holon is delegated to take part in this negotiation on behalf of the worker. For that, it consults the worker's AAS to get the information about the worker's capabilities and agenda. After the successful negotiation, it updates the worker's agenda with a new task allocation.

#### 5.3.2. Task configuration and execution

After the Resource Holons have committed to collaborate in carrying out the task, their skills are parameterized, if needed, and the task tree that represents the shared human-agent behavior is composed. The parameters for the skills are taken from the product AAS. The parameters for the worker's skills can choose, for example, different sets of instructions for the worker. For modeling of the shared task, a model called Behavior Trees (BTs) is used. The BTs have been chosen because they are easily composable into complex behaviors and are understandable by humans. A BT is a directed rooted tree with internal nodes called *control flow nodes* and leaf nodes called *execution nodes*. The control flow nodes set the rules on how to coordinate their child nodes. The execution nodes, *conditions and actions*, execute commands, which either change the agent's environment or check for changes in this environment. Further information about Behavior Trees can be found in Colledanchise and Ögren (2018). Sidorenko et al. (2022) showed in how BTs and skills from different devices can be composed together. As described in Figure 6, the holon has a set of behaviors that rely on the holon's skills. A behavior of the holon can be specified as a BT, where the BT's actions and conditions are realized by the holons's actions. The task BT is generated from the production step description taken from the product AAS. For the execution of the task BT, the Task Holon is spawned. Its goal is to connect to the BTs of the Resource Holons, i.e., the Human Digital Holon and the Handling Holon, and to coordinate their execution according to the logic of the task BT.

**Figure 10:**
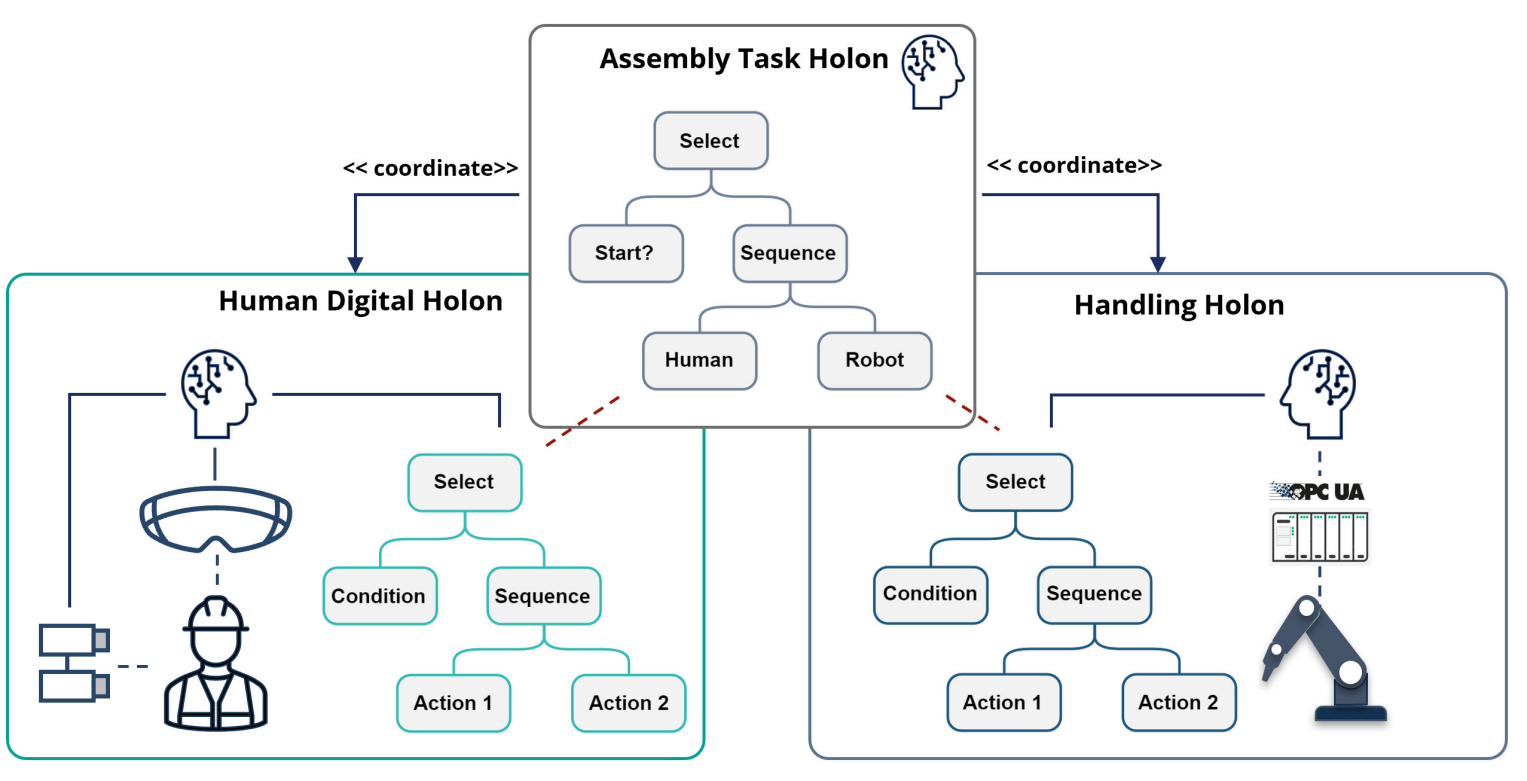
Shared task coordination by the Behavior Tree.

Figure 10 schematically shows the example of the shared assembly task coordinated by the BT. The actual BT of the task is more complex, but still is well understandable by the worker. The complete task BT can be projected on the worker's AR-glasses to provide the process overview. The BT gives the instructions to the worker through its actions. During the worker's actions, the Human Interface Holons observe the working scene and generate conditions to indicate that the current running action is completed.

#### 5.3.3. Implementation concept

**Figure 11:**
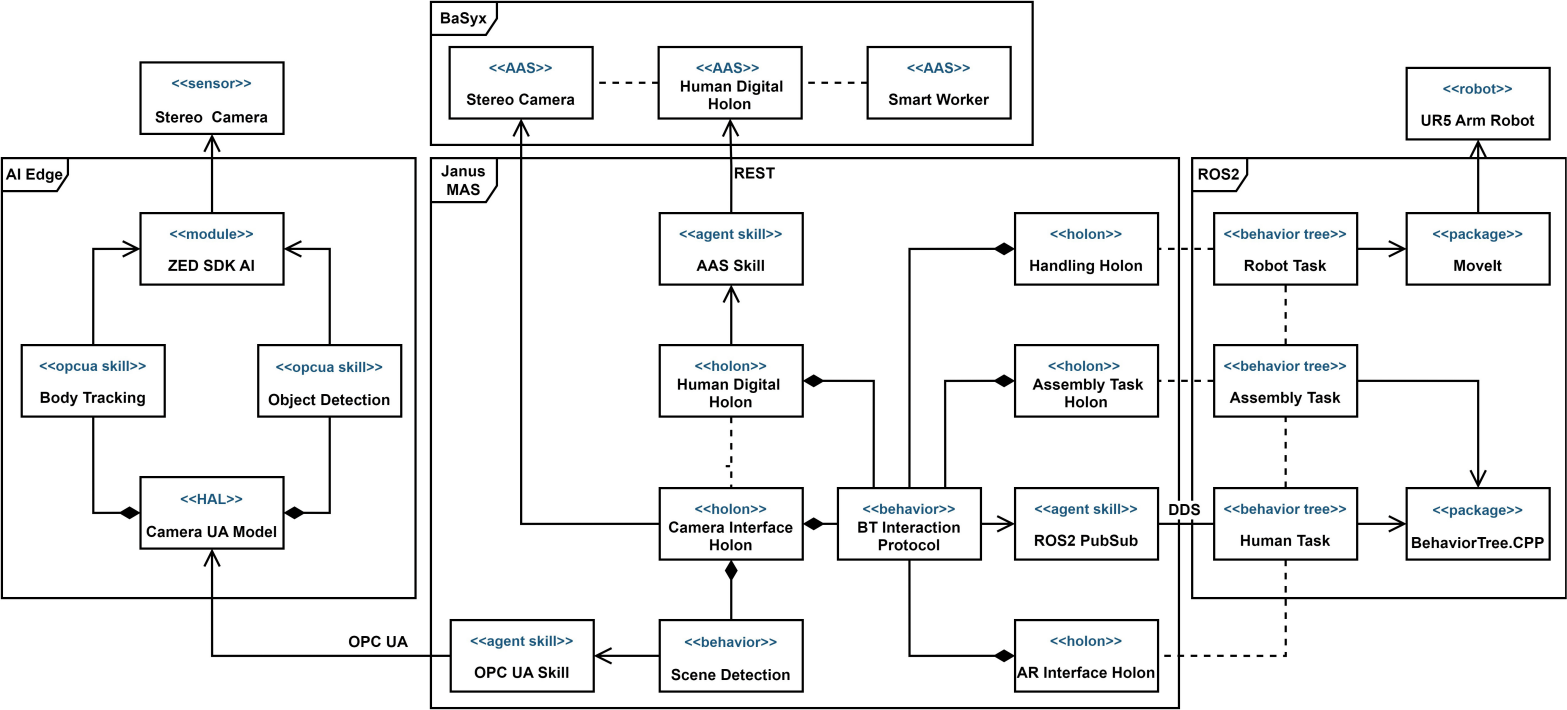
Block diagram showing the main components of the prototype concept.

This section provides further details on the implementation concepts of the previously discussed scenarios. Though the prototypes are still in the development phase, the main framework components have been implemented and reused from the previous scenarios. The block diagram in Figure 11 shows the main components of the prototype. There are four main subsystems:

- The stereo camera module for scene detection;
- The Robotic Operating System (ROS2) middleware for controlling the robot and executing Behavior Trees;
- The Janus runtime as the framework's MAS execution engine;
- The BaSyx middleware for hosting Asset Administration Shells and providing services to interact with them.

The stereo camera with the custom objects detectors and the hand tracking functionality is used to recognize changes in the assembly area. For the hardware configuration, we have chosen the ZED2 stereo camera^14^ and the Nvidia Jetson Orin Developer Kit^15^ because of the extensive capabilities of the ZED SDK AI module and the power of the Jetson Orin as an AI edge-device. The camera module is designed according to the principles discussed in Section 4.2. The OPC UA skills encapsulate and parameterize the detectors' algorithms and provide the standard skill interface (Volkmann et al., 2021). The ROS2 middleware will be used to control the UR5 robot with the MoveIt^16^ package. The BehaviorTree.CPP^17^ library will also be used to execute the Behavior Trees (BTs) of the assembly task. We have made this decision because there are no known Java realizations of the Behavior Trees execution engine. For our scenario, we have chosen the BehaviorTree.CPP library because of its maturity and integration with the ROS2. The interaction protocol for the synchronization of distributed BTs is described in Sidorenko et al. (2022). The protocol state machine is implemented as the SARL behavior. In Figure 11, the *Human Digital Holon* has a behavior *BT Interaction Protocol*, which uses a *ROS2 PubSub* skill. This skill realizes DDS^18^ publisher and subscriber for the communication with ROS2 nodes. A *Camera Interface Holon* tracks changes in the assembly working area and generates signals for the BT-conditions. It has a *Scene Detection* behavior, which uses the standard OPC UA skill to directly access the skills of the stereo camera module. Each holon also has an AAS skill, which uses the standard REST API of the AAS to retrieve all the required information about an asset (holon, CPPM, algorithm, etc.). In the current implementation, we use the BaSyx middleware for hosting AASs, but it is also possible to work with the different AAS platform or even with several ones by changing the AAS skill. Along with the BaSyx the Dimofac Digital Platform is being used in our project.

## 6. Discussion

This study tackles the topic of modular and reconfigurable production systems, where humans play an integral role and contribute to the adaptability and re-configurability of the system. The proposed methodology follows the RAMI4.0 reference architecture model and couples the skill-based/SOA approach with the holonic MAS, influenced by the ADACOR architecture (Leitão and Restivo, 2006). The AAS and standardization of the information models play the major role in ensuring interoperability between various components of CPPSs. The MAS framework has been extended to include human-machine interaction and consider both human and machine behaviors at the same level of abstraction. To achieve this, the human behavior was augmented with the digital holon.

The feasibility of the concept has been proven in the SmartFactory-KL^19^ on various industrial grade demonstrators, realizing the shared production environment based on the distributed testbeds, decomposition of production processes, agent-based production flow control, modular transportation systems and skill-based production modules (CPPM), as well as quality control components (Sidorenko et al., 2023). Furthermore, the testbed environment does also include the smart worker in its modular structure. Semantic technologies are used to model the human aspects as the submodels in the AAS, which in turn is used to create the Human Digital Holon, acting as the human digital twin. The Human Digital Holon allows the seamless integration and coordination of humans along with the other components of the digital system. In the MAS4AI project, the pilot lines in the real production environments are also equipped with the AI technologies such as semantic web, machine learning, and flexible planning, which are implemented as agents. This intends to show that the concepts are transferable to various kinds of production domains and industrial sectors. However, final evaluation is still open.

Two scenarios are described, which show how humans can be integrated into the shared human-machine tasks, and how this can be realized using the proposed framework. The first scenario shows the central role of the AAS and standardization in enabling plug-and-produce and self-configuration functionalities. The standardized submodels of the AAS provide all the information that the system requires for properly setting up an asset. The AAS serves as the standard self-description interface, providing the common information model and related services. Though, without standardization, it will be difficult to achieve. Agents rely on the standard information models to get the required information. As it can be seen from the activity diagram Figure 8, the implementation is simplified and serves as a proof-of-concept. Some functionalities have been left for further research and development. For example, Section 3.2 notices that a worker's role and capabilities depend on one's physical conditions and location in the production line. To realize such functionality, several Human Interface Holons together with the set of advanced sensors are needed. Moreover, to follow a human, the holons must be mobile, meaning they should be able to autonomously move from one MAS runtime to another. They will also need to acquire, change and share various sensors along the way. The holons will also need the AI algorithms to reliably track human conditions. This task is the research subject for the interdisciplinary teams. The second scenario shows the interplay of distributed and hierarchical approaches for designing control systems, which are separated in time. In the first phase, a distributed agent-based scheduling takes place, whereas in the execution phase the Task Holon uses the hierarchical Behavior Trees structure to orchestrate the skills of the scheduled holons. The holons temporary lose their autonomy and cooperate to accomplish the task. This allows to unify task allocation and execution at the agent level. The remarks from the first scenario are also valid here. Some questions have been left out of scope. For example, how to integrate low-level functional safety with the Behavior Trees or how to ensure reliable preemption of tasks? The Behavior Trees framework has already been established in the state-of-the-art robotics and gaming industry and offers several advantages in comparison with the more mature models, such as Hierarchical State Machines or Petri Nets. These are improved modularity, reactivity, extensibility, and explainability. Though, its further investigation is needed. Lastly, the Task Holon in the scenario acts like an orchestrator, which makes the solution more tightly coupled. Sidorenko et al. (2022) show how to execute the Behavior Trees in a distributed fashion, which enables to avoid the use of the Task Holon and execute a task similar to service choreographies.

While the human skills and competencies were adequately modeled in the AAS and used in the Human Digital Holon, the joint work of various interdisciplinary research groups is necessary to create the algorithms for the specialized Human Interface Holons. Furthermore, since human-related information is used, data privacy becomes important. The AAS has stated to support data security and privacy capabilities, but these aspects, to the best of the authors' knowledge, are still under development, and are not examined in the current study.

## 7. Conclusion and future work

In conclusion, this study proposes the MAS framework with the reference implementation for enabling the human-centered production paradigm. The concept allows seamless integration of smart workers with the MAS. Several challenging use-cases from a wide range of industrial sectors, including automotive, wood, bicycles, bearings, and metal, are involved to demonstrate scalability and flexibility of the framework for the deployment of AI solutions in different hierarchical layers of modular production. The pilot-lines are currently finalizing the deployment phase and starting gathering data for validation of the approach.

The main findings of this work can be described as follows. The combination of the RAMI4.0-based platform with the AASs can support the distributed manufacturing paradigm, where the AASs are used as data models for the smart software agents, which realize various AI algorithms and autonomously perform their tasks toward a joint goal. The RDF-based knowledge graphs can enhance information modeling capabilities of the AASs and support decision-making by the agents. Standardization of the AAS submodels is crucial for interoperability and plug-and-produce scenarios. Distributed planning and scheduling are necessary for modular reconfigurable architectures and increase flexibility and resiliency of the overall system, whereas hierarchical control structures can lower complexity and support efficient execution. Thus, the combination of holonic architectures with hierarchical behavior models, such as Behavior Trees, may be beneficial. By augmenting human behaviors with the digital holons and providing human related aspects to the system through the standard AAS submodels, an efficient human-machine integration can be achieved. Two scenarios have been presented to support the approach along with the concept for implementing the solution.

A formal evaluation of the architecture and the MAS prototypes will be a part of the future work. The evaluation will utilize the established methods, e.g., the Architecture Trade-off Analysis Method (ATAM).^20^ In addition, considering the need to diagnose failures in the MAS and ensure its predictability and robustness, formal methods will be used to evaluate its behavioral analysis using model-based techniques and simulation (Bos and Kleijn, 2002), as well as topological approaches with or without the integration of Fault-Detection-Isolation-Recovery techniques (Srivastava et al., 2020).

Finally, the next steps will target the expansion of the prototype with additional holons for solving planning and optimization tasks. The system's structure will also be refined to improve its performance concerning functional safety and real-time capabilities. Moreover, additional experimentation in the industrial case studies is required to examine the system's scalability and applicability in different industrial domains.

## Data availability statement

Publicly available datasets were analyzed in this study. This data can be found at: https://zenodo.org/communities/mas4ai/search?page=1&size=20.

## Author contributions

AS wrote the first draft of the manuscript. AS, WM, MB, and NN wrote sections of the manuscript. All authors contributed to the conception and design of the study, manuscript revision, read, and approved the submitted version.

## Footnotes

^1^ http://www.fipa.org/specs/fipa00023/XC00023H.html#_Toc526742625

^2^ https://www.plattform-i40.de/IP/Redaktion/EN/Downloads/Publikation/VWSiD%20V2.0.html

^3^ https://www.plattform-i40.de/IP/Redaktion/EN/Standardartikel/specification-administrationshell.html

^4^ https://www.bmwk.de/Redaktion/EN/Artikel/Digital-World/GAIA-X-Use-Cases/shared-production.html

^5^ https://esco.ec.europa.eu/en/about-esco/what-esco

^6^ https://www.onetcenter.org/overview.html

^7^ https://github.com/i40-Tools/RAMIOntology

^8^ http://www.fipa.org/repository/aclspecs.html

^9^ https://www.w3.org/TR/rdf-sparql-query/

^10^ https://eclipse.dev/basyx/?target=blank

^11^ https://dimofac.eu/

^12^ https://github.com/admin-shell-io/submodel-templates/tree/main/development/Asset%20Interface%20Description/1/0

^13^ https://kafka.apache.org/

^14^ https://www.stereolabs.com/zed-2/

^15^ https://www.nvidia.com/en-us/autonomous-machines/embedded-systems/jetson-orin/

^16^ https://moveit.ros.org/

^17^ https://www.behaviortree.dev/

^18^ https://design.ros2.org/articles/ros_on_dds.html

^19^ https://www.smartfactory.de/2022-production-level-4-oekosystem/

^20^ https://resources.sei.cmu.edu/library/asset-view.cfm?assetid=513908
